# Understanding Users’ App-Switching Behavior During the Mobile Search: An Empirical Study from the Perspective of Push–Pull–Mooring Framework

**DOI:** 10.3390/bs14110989

**Published:** 2024-10-24

**Authors:** Shaobo Liang, Ziyi Wei

**Affiliations:** 1 Research Institute of Digital Commerce, Wuhan Technology and Business University, Wuhan 430065, China; 2 School of Information Management, Wuhan University, Wuhan 430072, China; 3 Faculty of Education, University of Hong Kong, Pok Fu Lam, Hong Kong; weiziyi@connect.hku.hk

**Keywords:** mobile search, information search behavior, PPM model, switching behavior

## Abstract

With the rapid development of mobile applications (apps), various types of mobile apps have become the main channels for smartphone interaction. The user’s app switching behavior in mobile search tasks has also received attention from academia. This article uses the push–pull–mooring (PPM) theoretical model to determine the three influencing factors of push, pull, and mooring that affect user’s app switching behavior in mobile search. Data were collected from 374 respondents using a structural equation model. This study can deepen the understanding of app switching in user mobile search from the perspectives of information source selection, user information search path, etc. This study found that in terms of pushing factors, the complexity of search tasks positively affects users’ willingness to switch apps. In terms of pulling factors, the attractiveness of alternative products and users’ follow-up activities will positively affect their switching willingness. Meanwhile, inertia serves as a mooring variable to regulate the relationship between push-pull factors and user switching intentions. This research highlights key insights on user behavior, follow-up activities, and the role of switching costs and inertia, contributing to the broader literature on information-seeking behavior. It also provides actionable recommendations for app developers to enhance search experiences and retain users by integrating personalized, multi-modal features.

## 1. Introduction

With the widespread proliferation of smart mobile devices, search behaviors have increasingly gravitated towards mobile platforms. According to recent statistics, Google dominates the global mobile search engine market with a staggering 95.16% market share, reflecting the increasing reliance on mobile devices for search activities worldwide [1]. Mobile search activities are primarily conducted through three channels: search engines, web browsers, and specialized vertical applications.

As mobile search channels continue to evolve and become more sophisticated, users find it increasingly convenient to access information on mobile devices. However, the abundance of available information can lead to an “information overload” [2], creating challenges for users in effectively navigating and utilizing the data. As the global number of mobile users, highlighting the widespread reliance on mobile apps for various activities, including search, communication, and entertainment. In the face of numerous choices, users frequently switch between multiple apps during mobile searches to make informed decisions. For instance, users might compare product prices on different e-commerce apps like Amazon and eBay before making a purchase decision. The mobile app market has seen substantial growth, especially in dominant sectors like gaming and e-commerce. Furthermore, the large-scale release of apps allow users to navigate the vast array of available options [3].

Recognizing the vast market opportunities, internet companies have ventured into the mobile search sector. As a widely utilized online commercialization strategy, search engine optimization (SEO) has drawn substantial interest from advertisers. To some extent, understanding users’ search behavior equates to comprehending their usage preferences. Therefore, research on app-switching behavior can assist app developers in uncovering user habits and subsequently enhancing the functionality and user interface of their product to provide a more intuitive and personalized experience for users [4].

For a considerable period, many researchers have focused on searches using search engines. However, as mobile search diversifies, it is crucial to recognize apps as key platforms for these searches. Within mobile search research, there have been investigations into system usability [5], characteristics of cross-app search behavior [6], and search path extraction in mobile systems [7,8]. Despite the increasing diversity of mobile apps and their usage in search contexts, systematic studies on app-switching modeling remain underexplored. Traditional models have often focused on single-app usage patterns, overlooking the complexities associated with multi-app interactions and the factors driving users to switch between apps during mobile searches [9]. Recent research highlights that app-switching behavior is influenced not only by functional attributes of the apps themselves, such as usability and content relevance, but also by external factors like user context, situational triggers, and emotional states [4,10].

To address this gap, this paper employs the push––pull–mooring (PPM) framework—a well-established model in migration studies and consumer behavior research—as a theoretical foundation for examining the determinants of app transitions during mobile searches. The PPM framework is particularly suitable for this investigation because it accounts for a range of motivational drivers (“push” factors that encourage users to leave a current app), attractive alternatives (“pull” factors that draw users toward a different app), and situational or psychological constraints (“mooring” factors that moderate the switching behavior) [11,12]. Recent applications of the PPM framework in digital environments have demonstrated its robustness in explaining complex user behaviors, such as digital platform switching [13,14].

By integrating insights from these studies, this paper aims to provide a more nuanced understanding of the interplay between push factors, pull factors, and mooring factors influencing app-switching behaviors in the mobile search. In this study, we focused exclusively on mobile apps rather than mobile web searches conducted through browsers. While mobile web searches are prevalent, they fall outside the scope of this research, as we aim to assess app-specific behaviors. The application of the PPM framework allows for a comprehensive analysis that not only identifies key drivers of app transitions but also explores how different factors interact to shape user search paths and decision-making processes in a multi-app environment.

The subsequent sections of this paper are structured as follows: Section 2 provides a detailed literature review, highlighting the key theories and previous research. Section 3 introduces the research model and hypotheses. Section 4 outlines the research methodology, including the design and data collection methods. Section 5 presents the findings and analysis of the data, while Section 6 discusses the implications of the results and directions for future research.

## 2. Literature Review

### 2.1. Mobile Search Behavior Analysis

Research into mobile search began to emerge in the early 21st century. As smartphones became ubiquitous, scholarly attention increasingly shifted towards understanding mobile search behaviors. Kamvar and Baluja [15] conducted a comprehensive analysis of Google mobile search data, examining users’ search queries and the categories they frequently explored. In a subsequent study [16], they investigated post-query user behavior and discovered a critical insight: most users tend to abandon further exploration if their desired information is not found on the first page of search results. This finding underscores the importance of top-ranking search results in capturing user engagement, a trend that continues to be relevant in today’s mobile search environment.

Researchers have consistently focused on understanding the distinctive features of mobile search behavior. For instance, Church et al. [17] examined various aspects of mobile search, such as click-through rates, and emphasized the significant distinctions between mobile searches and traditional web searches. Their work highlighted how user engagement and interaction with search results vary substantially between these two platforms, owing to factors such as screen size and input methods. Similarly, Song et al. [18] conducted a comprehensive log-based analysis of user search behavior on mobile devices, including query categorization, query length, and the temporal distribution of search activities. Their findings underscore the need for search engines to adapt their algorithms and user interfaces to better accommodate the unique needs and constraints of mobile users.

With the rapid development of mobile search, the research directions are more diverse. For example, Guy [19] explored the semantic and syntactic features of voice search, shedding light on how natural language processing and user intent influence search outcomes in a mobile context. This work emphasizes the importance of understanding the nuances of spoken language, particularly as voice search becomes increasingly prevalent. Girod et al. [20] focused on the intricacies of content retrieval, highlighting the technical challenges and opportunities associated with recognizing and retrieving visual content on mobile devices, which requires sophisticated algorithms to handle varied image quality and contextual relevance.

Other scholars also focused on the consumer-driven aspect of mobile searches, where the demand for leisure-related information significantly shapes search patterns and user behavior [21]. Xie et al. [22] demonstrated the potential of mobile search systems to support a variety of query types—including images, audio, and video—highlighting the growing need for more versatile search engines capable of handling diverse forms of user input.

There are rich academic works from different perspectives on the topic of mobile search. For instance, when examining mobile search through a temporal lens, Amin posited that the immediacy of information needs often acts as a catalyst for initiating search behavior [23]. From a spatial standpoint, the context of the search environment significantly influences search themes; for example, users are more likely to search for food-related topics when located in a restaurant [7]. These findings suggest that both time and space are crucial factors that shape user search behavior on mobile devices.

### 2.2. App-Switching Behavior

In this study, “switching” is conceptualized not as a physical movement but as a form of digital migration. Wu et al. defines online transfer as the process whereby users reduce or cease the use of a particular technological product in favor of another that fulfills similar needs [24]. Similarly, Bansal and Taylor describe switching as the act of substituting one service provider with another [25]. Within this framework, app-switching behavior specifically refers to the transition of users from one mobile app to another during the mobile search process.

The literature on app-switching is extensive. Hu et al. provided a detailed examination of the behaviors involved in app-switching, identifying both shared and distinct motivations that drive these behaviors [26]. Chang et al. proposed a comprehensive classification of app-switching behaviors into three categories: switching from offline to online platforms, transitioning between similar service platforms, and switching between different types of platforms [27]. Research into these categories has revealed nuanced insights. Lai et al. focused on the first category, investigating the factors influencing the shift from offline to online services in elderly consultation projects [28]. On the other hand, Li utilized the push–pull–mooring (PPM) framework to analyze consumer behaviors related to switching from traditional membership cards to mobile apps [29]. For the second category, Gu et al. explored the phenomenon of “good-to-good” app-switching, drawing on consumer learning theory to understand why users transition between similar types of apps [30].

In exploring the third category, Cao examined the dynamics of users moving from blogging platforms to microblogging services like Weibo [31]. In addition, researchers have provided reflections from other perspectives. Although it does not explicitly explain why users switch, it proposes a comprehensive framework with clearly defined variables [32], which may help clarify the structure and relationships in the model.

This study focuses on the latter two types of app-switching behaviors identified in previous research. For example, a user might switch from Taobao to Temu for search purposes, representing a transition between two shopping apps. Alternatively, a user might move from a shopping app like Taobao to a social networking app, exemplifying a switch between different types of digital platforms. By analyzing these patterns of app-switching, the study aims to uncover the underlying motivations and contextual factors that drive such digital migrations in the mobile search context.

### 2.3. The Push–Pull–Mooring Framework

The push–pull–mooring (PPM) model originated in the study of human migration, where it has been a foundational theory in demography [33]. In demographic studies, migration is typically defined as the movement of individuals between two geographical locations over a certain period [34]. The conceptual foundations of the PPM framework were initially established with a focus solely on “push” and “pull” factors. Then, the “mooring” concept was integrated, forming the comprehensive PPM model used today. Within the PPM framework, “push” factors are seen as negative influences that drive individuals away from their current state or location, such as dissatisfaction or adverse conditions. Conversely, “pull” factors are positive attractions that draw individuals toward a new destination or state, like opportunities or benefits. “Mooring” factors, on the other hand, represent the constraints or obstacles that hinder the movement, such as emotional attachment or high switching costs [35].

The PPM framework has been widely applied beyond migration studies, finding relevance in various fields, including marketing, psychology, and technology adoption. For example, Hou et al. utilized the PPM model to examine how service quality in online gaming impacts user migration to other platforms [31]. Sun et al. adapted a modified version of the PPM framework to explore the factors influencing users’ switching behavior among mobile instant messaging apps [36]. Similarly, Hsieh et al. investigated the drivers prompting users to move between different online services, providing insights into the competitive dynamics of digital service markets [37].

Current research often employs dissatisfaction and the allure of alternatives to represent the “push” and “pull” components, respectively. This approach has been validated as effective in numerous studies. Fan and Suh argue that user dissatisfaction with a current system is a significant predictor of a switch to an alternative [38]. Similarly, Zengyan et al. consider dissatisfaction a critical explanatory variable when analyzing switching behaviors on social networking platforms [39]. Switching costs, frequently categorized as a “mooring” factor, are conceptualized and classified differently by various scholars. For instance, Nguyen et al. categorizes switching costs into program costs, financial costs, and relational costs [40]. In contrast, Maier et al. offer a different classification, identifying these costs as sunk costs, security costs, setup costs, and continuity costs [41].

This study builds upon well-established variables from existing scholarly research while also introducing novel variables to enhance the theoretical framework. For instance, dissatisfaction remains a critical component among the “push” factors, reflecting users’ discontent with their current situation or service. As an innovative addition, the “search task” is introduced to capture the specific objectives driving users’ search behaviors. Regarding the “pull” factors, the technology acceptance model (TAM) serves as a foundational reference [42], particularly emphasizing the perceived ease of use as a key determinant influencing users’ inclination to switch to a different platform or service. This dimension is crucial for understanding how the user-friendly nature of a new service can attract users away from their current choices. Within the context of “mooring” factors, this paper introduces a second-order construct, “inertia”, which represents the resistance to change due to habit or comfort with existing systems. To further enrich this concept, we incorporate innovative variables such as “technical self-efficacy”, which pertains to a user’s confidence in their ability to effectively use new technology. These variables are integrated into the hypothesis concerning switching costs, providing a more nuanced understanding of the factors that inhibit or facilitate switching behavior.

## 3. Research Model and Hypotheses

In this study, we adopt a more holistic approach by analyzing mobile search behaviors across a broad spectrum of users, rather than focusing on a specific demographic. Unlike prior research that has delved into specific facets of search behavior—such as search style, search timing, or frequency of search terms—we center our investigation on the phenomenon of app switching. Utilizing the push–pull–mooring (PPM) model, this paper aims to dissect the underlying motivations driving users to transition between different apps during mobile search process. By focusing on app-switching behavior, we seek to provide a comprehensive understanding of the factors influencing user navigation across multiple apps, thereby contributing to a more nuanced view of mobile search dynamics. Using the PPM framework as a guiding principle, we develop a set of hypotheses to predict app switching in mobile search.

### 3.1. Push Effects

#### 3.1.1. Dissatisfaction with Results

Within the domain of human migration studies, dissatisfaction is frequently identified as a primary catalyst prompting individuals to leave their current location [43]. Similarly, in the field of marketing, customer satisfaction plays a pivotal role in determining whether consumers will engage in repeat purchases [44]. Empirical evidence suggests that dissatisfaction with outcomes often heightens users’ propensity to seek alternatives. Research on the effects of satisfaction across various digital services, including email [45], blogging platforms [32], and web browsers [46], consistently demonstrates that high levels of satisfaction reduce the likelihood of users switching to different services. Conversely, dissatisfaction is more likely to drive a rejection of continued usage, directly influencing users’ decisions to switch. This perspective is further supported by Ref. [47]‘s findings, which indicate that dissatisfaction with social networking services (SNS) significantly enhances the intention to switch.

Given these insights, this study posits the following hypothesis:

**H1a.**  *Dissatisfaction with search results has a positive impact on the intention to switch between apps*.

#### 3.1.2. Search Task

In recent years, the contexts for mobile search have broadened considerably, reflecting a more generalized usage across diverse environments. Concurrently, the proliferation of specialized vertical search applications has introduced a wider range of complex search activities, such as learning-oriented searches [48] and exploratory searches [49]. Much research has examined the relationship between the nature of search tasks and corresponding search behaviors. For example, Wei and Zhang, through an analysis of search logs, demonstrated that as the complexity of search tasks increases, search behaviors tend to become more exploratory and comprehensive [50]. Additionally, Reisoğlu et al. established that task complexity significantly influences information-seeking behavior, leading to more varied and extensive search patterns [50].

These findings from prior research suggest that the complexity inherent in search tasks can impact app-switching behavior, with more intricate tasks often fostering a greater inclination to switch apps. This aligns with the notion that users, when confronted with complex or multifaceted tasks, may exhibit a stronger intention to move between different apps to fulfill their information needs.

Based on these insights, this study proposes the following hypothesis:

**H1b.**  *Search tasks have a positive impact on the intention to switch between apps*.

### 3.2. Pull Effects

#### 3.2.1. Alternative Attractiveness

Previous research has consistently demonstrated that the perceived alternative attractiveness significantly influences users’ intention to switch apps. For instance, Zengyan et al. highlighted that when users perceive the essential features of an alternative service to be superior, they are more inclined to shift their preference towards it [39]. In a comprehensive theoretical analysis combined with empirical testing, Bhattacherjee et al. also established that the relative advantages offered by alternative options positively correlate with the intention to switch [51]. Similarly, in their investigation of mobile shopping behaviors, Lai et al. found that users, driven by the desire for a superior shopping experience, tend to utilize multiple channels, including group discussions, voting mechanisms, or information exchanges [52]. When a particular channel offers more substantial advantages as a substitute, users are naturally drawn to it.

These findings collectively suggest that the comparative benefits of alternative attractiveness play a critical role in shaping user behavior, particularly in contexts where switching costs are relatively low, and users are continuously seeking better experiences. Understanding these dynamics is crucial for service providers aiming to retain users by enhancing the perceived value of their offerings relative to competitors.

The contemporary app market is vast, offering users an increasingly diverse range of app choices. For instance, if users perceive that a shopping app like “Poizon” offers more specialized recommendations and curated product selections, they are more inclined to migrate from established platforms such as Taobao to Poizon. This behavior illustrates how differentiation drives user decisions to switch from one app to another.

Drawing upon existing theoretical frameworks, this study proposes the following hypothesis:

**H2a.**  *The alternative attractiveness has a positive impact on the intention to switch between apps*.

#### 3.2.2. Follow-Up Activities

Empirical observations suggest that users’ needs for actions following an initial search can significantly influence their likelihood of switching between apps. For instance, a user who finalizes their travel plans after exploring tourism landscapes and reading travel reviews on a dedicated travel app might subsequently use a weather app and a navigation app to develop specific travel strategies. Böhmer et al. discovered that users are most likely to engage with instant messaging apps immediately after completing a mobile search [53]. This indicates that, despite limited research on “follow-up behavior”, some scholars have begun to recognize its effect on mobile search dynamics. Previous research suggests that follow-up activities, such as engaging with related content, can increase user retention and reinforce learning outcomes [4,6,7]. This body of work aligns with the hypothesis that follow-up activities enhance user interaction by providing continuous value after the initial search, which may lead to higher levels of user satisfaction and app engagement.

Building on previous findings and contextual observations, this study proposes the following hypothesis:

**H2b.**  *Follow-up activities have a positive impact on the intention to switch between apps*.

#### 3.2.3. Perceived Ease of Use

The technology acceptance model (TAM) was initially introduced by Davis [54], positing that user acceptance of a new technology is primarily influenced by two critical factors: perceived ease of use and perceived usefulness. Within the TAM framework, perceived ease of use is a central construct that reflects the extent to which an individual believes that engaging with a particular technology or system will be free from effort. This concept suggests that the simpler and more intuitive a system is perceived to be, the more likely users are to adopt and integrate it into their regular routines. By emphasizing the importance of perceived ease of use, TAM underscores how user-friendly interfaces and straightforward functionalities can significantly enhance the likelihood of technology acceptance, thereby shaping user behavior and technology diffusion patterns in various contexts.

Currently, the concept of perceived ease of use is extensively applied across diverse disciplines, including marketing, psychology, and technology adoption studies. In examining app-switching behavior, Roy identified that perceived ease of use significantly influences users’ choices when selecting among various apps [55]. Similarly, Indarsin and Ali concluded that perceived ease of use positively shapes user attitudes toward adopting m-commerce platforms [56]. Furthermore, in their research on the switching behaviors associated with short video apps, perceived ease of use substantially affects users’ intentions to switch between apps [57]. Drawing on these findings from the existing literature, this paper conceptualizes perceived ease of use within the context of mobile search as users’ perception of the effort required to utilize different search apps.

Accordingly, this study proposes the following hypothesis:

**H2c.**  *Perceived ease of use has a positive impact on the intention to switch between apps*.

### 3.3. Mooring Effects

In digital migration studies, the concept of mooring factors serves as a moderating element that influences the relationship between push and pull factors and the ultimate decision to migrate [43]. Rather than directly driving or attracting individuals, mooring factors act as constraints or enablers that shape the decision-making process. For instance, it was suggested that mooring factors do not directly motivate or deter consumers [52]. Instead, they play a critical role in influencing the decision to adopt mobile shopping by modulating the interplay between push factors, such as dissatisfaction with existing options, and pull factors, like the perceived benefits of new alternatives. By mediating the effects of these opposing forces, mooring factors help to fine-tune the decision-making process, allowing for a more comprehensive understanding of how users navigate their choices in complex environments.

#### 3.3.1. Inertia

Samuelson and Zeckhauser found that individuals have a propensity to prefer maintaining their current state or behaviors when faced with decision-making scenarios [58]. Inertia, a manifestation of this bias, originally comes from a physical science definition, referring to the tendency of an object to remain in its state of motion unless acted upon by an external force [59]. In psychological contexts, Polites and Karahanna conceptualize inertia as a tendency for individuals to stick with existing habits and reduce their adoption of new technologies [60].

Building upon existing studies, it is evident that users are often influenced by inertia. This tendency may cause users to refrain from switching to new products, even if their current tools are suboptimal, due to their emotional attachment and familiarity with the existing ones. Inertia can diminish an individual’s cognitive assessment of the perceived ease of use and the comparative advantages of alternative systems, thereby negatively impacting their intention to adopt new technologies. Consequently, inertia can be viewed as a deterrent to switching behavior.

In this study, inertia is dissected into three distinct dimensions: behavioral, cognitive, and emotional. Behavioral inertia pertains to the continuation of using a service simply because it is habitual. Cognitive inertia involves a conscious decision to stick with the current service, despite recognizing that it may not be the most optimal choice. Emotional inertia reflects the emotional attachment or preference for the current service, leading to its continued use. In the context of mobile search, users are likely to favor their existing apps because inertia leads them to perceive newer alternatives as less appealing. Essentially, inertia reduces the likelihood of switching intentions, even when users are aware of the potential benefits of alternative options.

Based on this understanding, the study posits the following hypothesis:

**H3.**  *Inertia has a negative impact on the intention to switch between apps*.

#### 3.3.2. Antecedents of Inertia

(1) Switching Cost

Current research on mooring factors primarily examined cognitive aspects, such as switching costs [61]. Scholars offer varying definitions and classifications of switching costs. For instance, Burnham et al. categorized switching costs into three types: procedural, financial, and relational [62]. This study focuses initially on cognitive switching costs, which encompass users’ perceptions of the time, effort, and expenses required to learn and adapt to new interfaces or channels when switching [63]. Hou et al. highlighted that users often need to invest additional time and energy to become familiar with new services [64]. In his examination of user behavior on B2C websites, Shih suggested that developing skills and habits in unfamiliar environments inevitably incurs cognitive adaptation costs [65]. Pourabeddin et al. argued that high cognitive switching costs can act as a significant barrier to switching, deterring consumers from transitioning to alternative options [66].

Beyond cognitive costs, research also explores other dimensions of switching costs. For example, Ye and Potter identified new challenges that users encounter when engaging in e-commerce [67]. Wu and Wang contended that equipment costs, access fees, and transaction costs are crucial factors that make mobile commerce more expensive than traditional wired e-commerce [68]. Generally, these expenses are framed as setup costs, continuity costs, and learning costs, all of which contribute to reducing consumers’ willingness to switch to mobile shopping platforms. Consequently, switching intentions are heavily influenced by both switching costs and the level of trust in the new service.

In summary, this paper posits that cognitive switching costs, learning costs, and financial costs contribute to user inertia. Therefore, the study hypothesizes:

**H4a.**  *Switching cost has a positive impact on inertia*.

(2) Prior Habit

The concept of habit is rooted in psychology. Drawing on the notion of Status quo bias, Polites and Karahanna describe habit as a series of learned behaviors that have become automatic responses to specific stimuli [60], aimed at achieving particular objectives or end states. Ye and Potter further suggest that habitual behaviors can negatively impact an individual’s willingness to switch to alternative options [67]. In the context of online services, users who have grown accustomed to a particular service are less likely to engage in a thorough comparison of the advantages offered by different alternatives. Instead, they tend to rely on automatic, habitual responses, reinforcing their existing behavioral patterns.

Habitual behavior, as a form of automatic reaction, helps individuals minimize the cognitive effort involved in decision-making processes. Moreover, for users who are averse to the discomfort associated with change, engaging in habitual actions that demand minimal cognitive resources becomes a preferred choice. Such users are more inclined to maintain their current behaviors and resist change, thereby preserving the status quo [69]. This analysis suggests that habit serves as a cognitive shortcut, allowing users to avoid the perceived costs and uncertainties associated with exploring new options, thus reinforcing continuity in their current usage patterns.

In this paper, the current habits mean that users are used to using existing apps. Based on it, this paper hypothesizes that:

**H4b.**  *Prior habit has a positive impact on inertia*.

(3) Technical self-efficacy

Self-efficacy refers to an individual’s belief in their own capabilities to execute tasks and achieve goals [70]. The fundamental premise is that one’s perceived self-efficacy, shaped by cognitive assessments, influences both behavior and the willingness to invest effort [71]. Technical self-efficacy can be considered a specific subset of this broader concept, representing an individual’s confidence in their ability to effectively and accurately utilize a particular technological tool or product.

Building on the elaboration likelihood model (ELM), Zhou explored how information self-efficacy moderates the central and peripheral routes of information processing in the context of digital libraries [72]. Their findings highlight that higher levels of self-efficacy enhance users’ ability to engage more deeply with information, influencing their information-seeking behaviors. Similarly, Torres and Gerhart investigated the role of information self-efficacy in shaping perceived ease of use within the domain of information terminals, demonstrating its positive effect on users’ technology adoption [73]. Zhang et al. further discovered that individuals with lower levels of self-efficacy are more concerned with privacy, suggesting a cautious approach to new technologies [74].

In the context of mobile search apps, this study posits that users with higher technical self-efficacy are more confident in their ability to master new search functionalities and navigate unfamiliar interfaces. Consequently, they are less likely to exhibit inertia, or resistance to switching to new apps, due to their confidence in learning and adapting to new systems. Thus, this study hypothesizes:

**H4c.**  *Technical self-efficacy has a positive impact on inertia.*

**Figure 1:**
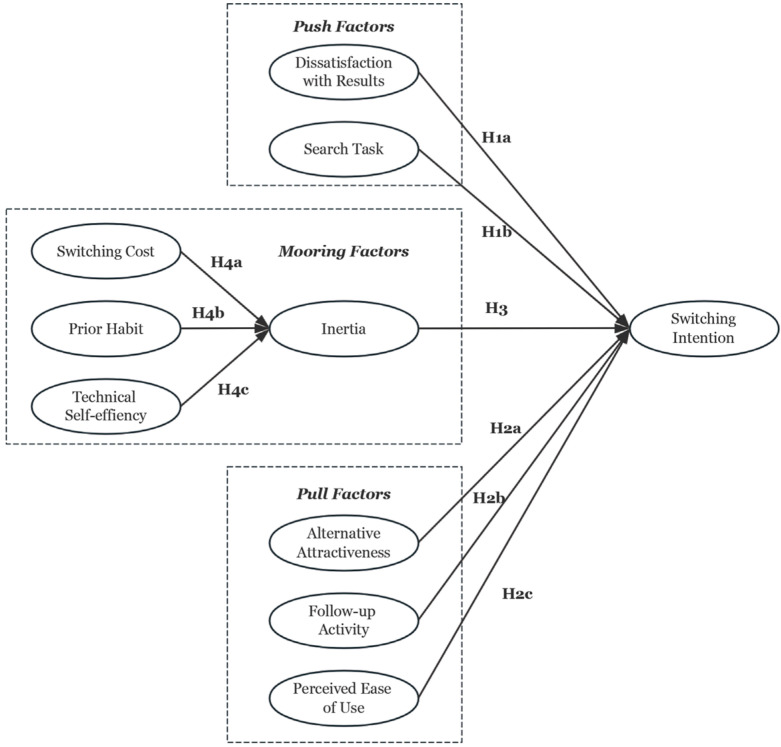
Research Model.

The research model proposed in this paper is illustrated in Figure 1.

## 4. Method

### 4.1. Data Collection

This study employed an online survey-based data collection method, utilizing a structured questionnaire to gather information from participants. The questionnaire consisted primarily of closed-ended questions measured on a Likert-type scale to quantify responses. Initially, we distributed 435 preliminary questionnaires aimed at gathering background information on participants, including their daily mobile search activities and experiences with app switching. We select formal respondents in the preliminary questionnaire by asking users if they have any app-switching experience. At the same time, in order to ensure that the respondents fully understand the research context and concepts, we provided specific examples to help them understand the scope of this study.

To ensure that the questionnaire accurately captured responses specific to search apps, an introductory text was provided before respondents began the survey. This introduction clarified that the focus of the study was on search apps, defining the scope of the questions. Additionally, each set of questions included specific context to further emphasize that the research was examining user behavior in the context of search apps. These steps were taken to reduce confusion and ensure that respondents understood the purpose of the study.

After refining the questionnaire and selecting suitable respondents based on initial feedback, a total of 374 responses were collected. Subsequently, we conducted a reliability analysis to ensure data integrity, which led to the exclusion of 42 responses that did not meet the required reliability criteria. This process resulted in a final dataset of 332 complete and valid questionnaires for further analysis.

### 4.2. Measures

The questionnaire was structured into two sections. The first section focused on collecting demographic information and data regarding the number of apps used, as detailed in Table 1. The second section comprised questions designed to explore app-switching behaviors in the context of mobile search. All constructs included in the survey were measured using items previously validated in the academic literature, with slight adaptations made to align them with the specific objectives and context of this study.

The questionnaire items, outlined in Table 2, were evaluated using a 7-point Likert scale ranging from 1 (strongly disagree) to 7 (strongly agree), allowing for a nuanced assessment of respondents’ attitudes and behaviors.

Prior to conducting hypothesis testing, we conducted a thorough assessment of the reliability and validity of the construct measurements. To evaluate the reliability of the scales used, we employed several statistical measures, including average variance extracted (AVE), composite reliability (CR), and Cronbach’s alpha. The threshold values for these indicators are well-established in the literature, with AVE and CR requiring minimum values of 0.5 and 0.7, respectively, and a recommended cutoff of 0.7 for Cronbach’s alpha.

As presented in Table 3, the lowest values recorded for AVE, CR, and Cronbach’s alpha were 0.521, 0.842, and 0.742, respectively. Each of these values exceeds the suggested thresholds, indicating that all the constructs measured in the study exhibit a high degree of reliability. This robust reliability suggests that the items used in the constructs consistently capture the underlying theoretical concepts intended for measurement, ensuring the soundness of the subsequent data analysis.

The validity of the construct measurements can be assessed through factor analysis, which determines whether the classification of the scale’s structure is appropriate. For factor analysis to be meaningful, there should be a strong inter-item correlation, typically evaluated using two key metrics: the Kaiser–Meyer–Olkin (KMO) measure and Bartlett’s Test of Sphericity. In this study, the KMO value was found to be 0.901, and Bartlett’s Test of Sphericity indicated statistical significance (*p* < 0.001), confirming the suitability of the data for factor analysis.

Using principal component analysis (PCA) with varimax rotation, 8 factors with eigenvalues exceeding 1 were extracted, accounting for 75.179% of the total variance. This high cumulative explained variance suggests that the scale possesses robust validity, effectively capturing the constructs intended for measurement.

Discriminant validity was further evaluated using a correlation matrix and the square roots of the average variance extracted (AVE) for the primary constructs. According to established criteria, the square root of the AVE for each construct should exceed its correlations with all other constructs in the model to confirm discriminant validity. As shown in Table 4, the square roots of all AVEs were indeed greater than the correlations with other constructs, providing evidence of adequate discriminant validity. This indicates that each construct is distinct and uniquely contributes to the overall model.

## 5. Results

**Figure 2:**
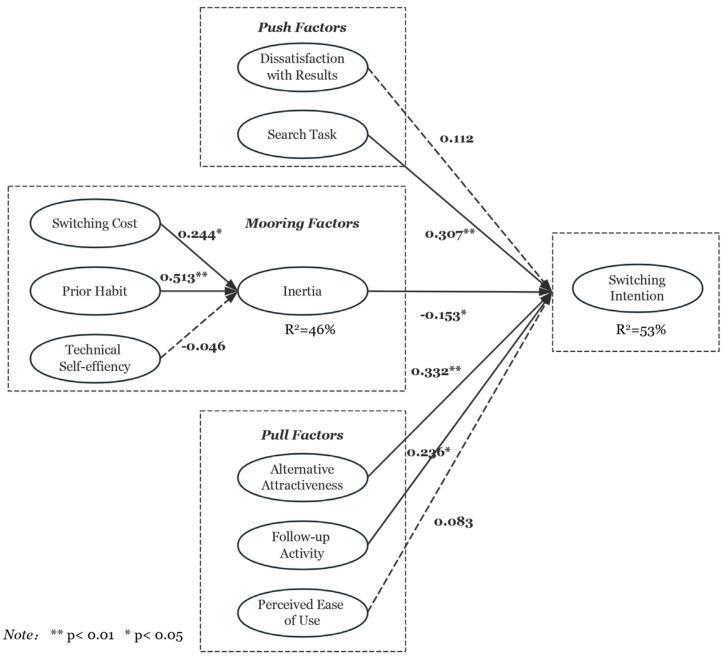
Results of structural model.

Following the confirmation of satisfactory reliability and validity of the measurement constructs, hypothesis testing was conducted using the research sample. The analysis was performed using SmartPLS 4.1.0.8 software, which facilitated the structural equation modeling (SEM) process. Through this approach, key metrics—including path coefficients, *p*-values, t-values, and the explained variance (R^2^) for each hypothesis—were derived from the path analysis. These results provide a comprehensive understanding of the relationships between the constructs. Detailed results and statistical outputs are presented in Figure 2.

### 5.1. Summary of Findings

In this study, a significance level of 0.05 was established as the threshold for determining statistical significance. In push and pull factors, the path coefficients for search task, alternative attractiveness, and follow-up activities were found to be 0.307, 0.332, and 0.236, respectively. Each of these coefficients was statistically significant, with *p*-values less than 0.01, 0.01, and 0.05, respectively.

These findings indicate that, as hypothesized, search task, alternative attractiveness, and follow-up activities exert significant positive influences on app-switching intention. Thus, the data support hypotheses H1b, H2a, and H2b, demonstrating that these variables are key determinants in shaping users’ propensity to switch between applications.

The results align with the existing literature, which suggests that both the perceived benefits of alternative options and the specific characteristics of user tasks and behaviors play crucial roles in driving switching behavior in digital environments.

For the mooring factors, the path coefficients for switching cost and prior habit were found to be 0.244 and 0.513, respectively, both showing statistical significance (*p* < 0.05 and *p* < 0.01). These results confirm the hypothesized positive relationships between switching cost, prior habit, and inertia, thereby supporting H4a and H4b. Additionally, the path coefficient for inertia was −0.153 (*p* < 0.05), providing evidence in favor of H3, which proposed a negative relationship between inertia and switching intention.

However, contrary to expectations, the effects of dissatisfaction with results, perceived ease of use, and technical self-efficacy were only marginal, leading to the rejection of H1a, H2c, and H4c. The explanatory power of the model was assessed using the R^2^ values. Inertia and switching intention had R^2^ values of 46% and 53%, respectively, indicating that the model provides a sufficient level of explanation for the variance in switching behavior.

### 5.2. Proven Factors of Switching Intention

The findings indicate that when users engage in complex search tasks, they often find it challenging to obtain the desired results from a single platform. As a result, many opt to use multiple apps to complete their search objectives. In light of this, app developers should recognize the diverse needs of users and avoid concentrating solely on singular user requirements.

The study also confirms that the attractiveness of alternative apps positively influences switching intentions during mobile searches. As the app market continues to grow, users are increasingly open to switching to new platforms that offer superior search experiences. Thus, maintaining a competitive edge should be a strategic priority for developers.

Follow-up activities fall into two primary categories. The first involves continuing the search process, such as searching for reviews on social media after gathering product details. The second category consists of non-search actions, like sharing information on platforms such as WeChat after completing a search. App operators should place greater emphasis on these follow-up behaviors, as they are known to drive switching intentions.

In contrast, inertia exerts a negative effect on switching intentions. When users develop emotional, cognitive, and behavioral attachments to a particular app, switching to another platform may be perceived as burdensome. Moreover, the results reveal that switching costs and entrenched habits positively contribute to inertia. These insights suggest that when attempting to attract users from other platforms, app operators should focus on reducing switching costs and facilitating a change in users’ established habits.

## 6. Discussion

### 6.1. Practical Implications

For practitioners, this study delivers practical guidance on how to thrive in an increasingly competitive app market. First, mobile search plays a pivotal role in driving user engagement and should be treated as a core component of the user experience. As competition intensifies, app developers must focus on improving search functionalities. This can be achieved by analyzing user search patterns—including search queries, click behavior, and content preferences—and integrating these insights into advanced recommendation systems based on user profiling. These systems can offer personalized search results that anticipate user needs, improving both satisfaction and retention.

Moreover, presenting search results in varied formats, such as images, infographics, videos, and interactive elements, can significantly enhance user engagement. This diversity not only caters to different learning styles but also helps maintain user interest, reducing the likelihood of switching to competing platforms.

As mobile search platforms expand into specialized fields like e-commerce, social networking, and healthcare, there is a risk of functionality overlap making it difficult for individual platforms to stand out. Furthermore, the complexity of search tasks often means that users cannot rely on a single platform to meet all their needs. Users frequently turn to additional apps for follow-up actions—such as product reviews or social media sharing—highlighting the need for integrated services.

Mobile search platforms should therefore explore opportunities for offering holistic, one-stop solutions that reduce the need for app-switching. By integrating various services and search scenarios within a single platform, developers can lower switching costs and offer users a more seamless experience. This not only boosts retention but also enhances user loyalty, providing a long-term competitive advantage in the crowded app market. Furthermore, collaborations with external platforms or cross-platform functionalities could be explored, allowing users to access a wider array of services without leaving the primary app, thus fostering a more comprehensive ecosystem.

Lastly, as behavior switches toward more dynamic and multifaceted search processes, platforms must continue to innovate, ensuring they remain agile and responsive to evolving consumer expectations. Strategic partnerships, AI-driven innovations, and continuous user feedback loops will be essential in adapting to these shifts, helping platforms maintain their relevance and competitive edge.

### 6.2. Limitations and Future Work

This study presents some limitations that future research could address. First, the definitions of certain variables, such as follow-up behavior, require further clarification due to the lack of existing literature, resulting in uncertain responses in the questionnaire. Additionally, classical variables used in previous research may not be fully applicable in app-switching contexts, suggesting the need for more contextually relevant questions in future surveys. Lastly, our sample was primarily drawn from respondents in the manufacturing industry, limiting the diversity of our data, which should be expanded in future research.

We also recognize the potential need to refine our model by removing less significant variables and exploring new factors. For instance, future work may introduce variables better suited to app-switching behavior or behavioral patterns that have stronger theoretical relevance, such as hedonic motivation in different search contexts.

In this study, we measured switching intention as a proxy for actual switching behavior, which may not fully capture the real-world actions users take. While perceived switching intention reflects users’ inclination to switch apps, it is essential to differentiate between intention and actual behavior. Future research should examine the relationship between these two variables to clarify the extent to which switching intention translates into actual switching behavior. This can be achieved by incorporating behavioral tracking data to monitor real app-switching patterns and usage over time. This enhancement would provide deeper insights into the determinants of both intention and subsequent behavior in app-switching contexts.

## 7. Conclusions

This study explored the factors influencing app-switching behavior in mobile search environments, using the push–pull–mooring framework. The findings confirm that search task complexity, alternative attractiveness, and follow-up activities positively impact switching intentions, while inertia acts as a deterrent. Interestingly, traditional variables like dissatisfaction and perceived ease of use showed limited relevance in this context, suggesting that app-switching requires more context-specific considerations. Practical implications include the importance of improving user experience through integrated services and reducing switching costs.

## Figures and Tables

**Table 1:** Demographic statistics.

		Number	Percentage (%)
**Gender**	Male	159	47.9
	Female	173	52.1
**Age**	<18	1	0.3
	18–25	65	19.6
	26–30	98	29.5
	31–35	51	15.4
	36–40	57	17.2
	>40	60	18.1
**Education**	High school or below	76	22.9
	Bachelor’s degree	189	56.9
	Master’s degree and above	67	20.2
**Occupation**	State organs	35	10.5
	Manufacturing	108	32.5
	Education	63	19.0
	Student	46	13.9
	Self-employed	68	20.5
	Other	12	3.6
**The number of apps on mobile phones**	<20	126	38.0
	21–30	106	31.9
	31–40	64	19.3
	>40	36	10.8

**Table 2:** Measurement items.

Construct	Identifier	Item
**Dissatisfaction with results** (Bansal, 2005 [25]) (Lai, 2012 [52])	DWR1	I am not satisfied with my experience using my current mobile app.
	DWR2	I feel very angry with my experience using my current mobile app.
	DWR3	I feel very unhappy about my experience using my current mobile app.
	DWR4	I feel very terrible about my experience using my current mobile app.
**Search task**	ST1	The current app can’t help to complete the search task.
(Vakkari P, 2016 [48])	ST2	I need another app to continue my search task.
	ST3	In general, a single app can’t complete my search task.
**Alternative attractiveness** (Bhattacherjee, 2012 [51]) (Lai, 2012 [52])	AA1	If I need to change to another mobile app, there are other better options.
	AA2	I might be more satisfied with another app’s functions and service.
	AA3	There are other more satisfying apps compared with the current one.
**Follow-up activity** (Böhmer M, 2011 [53])	FA1	I will continue follow-up activities on other apps after the initial search.
	FA2	The current app can’t help me with more follow-up activities.
	FA3	If I want to continue follow-up activities, I will change to another app.
**Perceived ease of use**	PEU1	Interactions with apps don’t need much mental work.
(Roy, 2017 [55])	PEU2	I found apps are very easy to use.
	PEU3	I found it easy to do things I want with apps.
**Inertia**		
Emotional-based	EBI1	I will continue using my current app because changing it gives me pressure.
	EBI2	I will continue using my current app because I am willing to do it.
	EBI3	I will continue using my current app because I like to do it.
Behavior-based	BBI1	I will continue using my current app because I do it all the time.
	BBI2	I will continue using my current app just because it is a part of my daily life.
	BBI3	I will continue using my current app because I used to do it.
Cognitive-based	CBI1	I will continue using my current app although I know it is not the best way.
	CBI2	I will continue using my current app although I know it is not the most efficient way.
	CBI3	I will continue using my current app although I know it is not the most effective way.
**Switching cost** (Hou, 2011 [64]) (Shih, H.P, 2012 [65])	SC1	In general, switching to other apps will be trouble.
	SC2	Switching to other apps will cost much time and energy
	SC3	If I switch to other apps, I will lose a lot.
**Prior habit**	PH1	Selecting my current app is an automatic behavior.
(Polites and Karahanna, 2012 [60])	PH2	It is natural for me to use my current app.
	PH3	It is an obvious choice to use the current app when I search for information.
**Technical self-efficacy** (Zhang X, 2018 [74])	TS1	I can easily switch to another app if I want.
	TS2	I can use the functions of a new app, even if no one teaches me.
	TS3	I will be able to use the functions of a new app skillfully.
**Switch intention**	SI1	I am considering switching from my current mobile app
(Kim et al., 2006 [45])	SI2 SI3	?The chance of my switching to another mobile app is high. I am determined to switch to another mobile app

**Table 3:** Reliability.

Construct	Cronbach’s Alpha	CR	AVE
DWR	0.956	0.968	0.883
FA	0.742	0.842	0.642
PH	0.876	0.924	0.802
IN	0.880	0.905	0.521
PEU	0.835	0.896	0.742
TS	0.859	0.907	0.765
ST	0.886	0.930	0.815
AA	0.843	0.897	0.744
SC	0.913	0.945	0.852
SI	0.942	0.963	0.896

**Table 4:** Correlation matrix and square roots of AVE.

	DWR	FA	PH	IN	PEU	TS	ST	AA	SC	SI
DWR	0.940									
FA	0.381	0.801								
PH	0.215	0.072	0.896							
IN	0.302	0.163	0.596	0.722						
PEU	0.045	0.329	0.081	0.055	0.862					
TS	0.121	0.280	0.155	0.092	0.472	0.875				
ST	0.652	0.462	0.068	0.131	0.107	0.213	0.903			
AA	0.227	0.373	0.125	0.182	0.519	0.592	0.300	0.863		
SC	0.489	0.189	0.312	0.398	−0.082	−0.135	0.411	0.016	0.923	
SI	0.395	0.392	−0.040	0.124	0.226	−0.015	0.488	0.329	0.337	0.947

## Data Availability

The research data can be accessed upon request to the corresponding author.
